# A Young Woman Presenting with Encephalopathy: A Case Report

**DOI:** 10.1007/s12028-019-00872-8

**Published:** 2019-11-01

**Authors:** Mario Kofler, Stefan Kiechl, Raimund Helbok

**Affiliations:** Department of Neurology, Medical University of Innsbruck, Anichstrasse 35, 6020 Innsbruck, Austria

**Keywords:** Susac syndrome, Encephalopathy, Magnetic resonance imaging, White matter lesions, Differential diagnosis

## Introduction

When cerebral white matter lesions are detected in young patients presenting with neurologic symptoms, the most likely diagnosis is multiple sclerosis (MS). Still, it is crucial to screen for important differential diagnoses which require sophisticated workup and sometimes immediate treatment, such as autoimmune encephalopathies, juvenile stroke, central nervous system (CNS) vasculitis, and, as in this case, Susac syndrome. Knowledge about key clinical and neuro-imaging features is therefore of utmost importance. Here, we report a case of a young woman presenting with encephalopathy, focal neurological deficits, and cerebral white matter lesions and discuss our diagnostic approach.

## Case

**Fig. 1:**
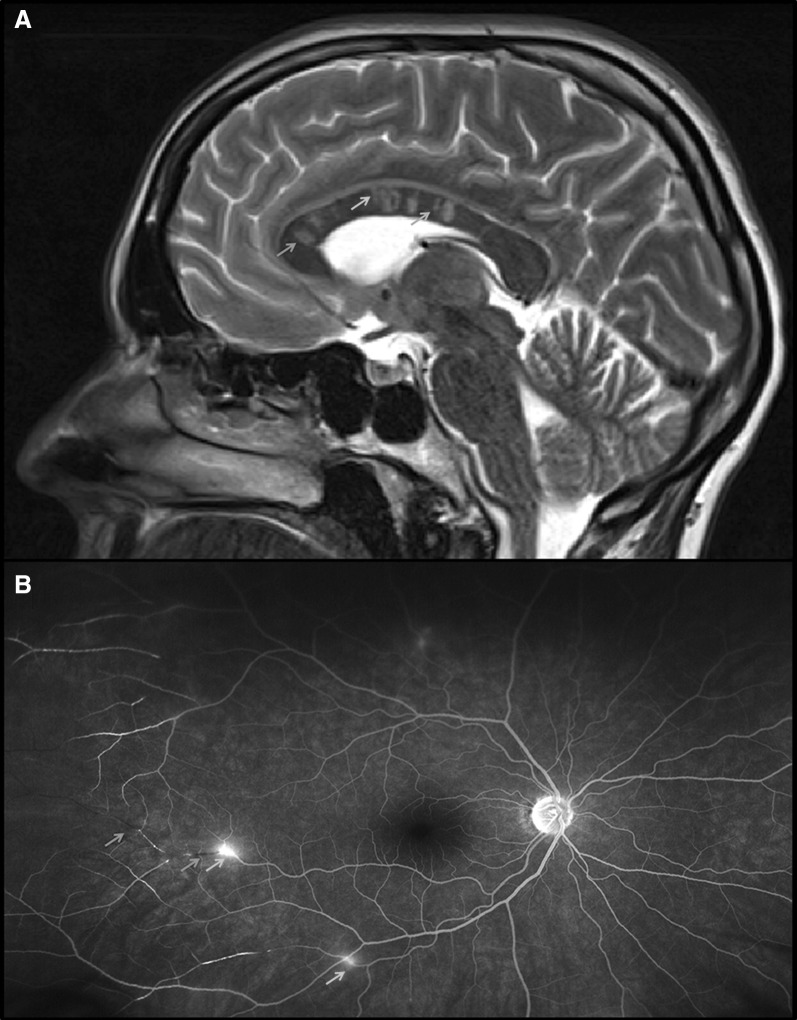
**a** T2-weighted sagittal MRI scan of the brain showing round (*snowball,* yellow arrow), “hanging” (*icicle,* red arrow) and linear (*spoke,* blue arrow) hyperintense lesions in the central corpus callosum; **b** fluorescein angiography of the retina revealing branch retinal artery occlusions (yellow arrows) and arterial wall hyperfluorescence (red arrows) (Color figure online)

A previously healthy 30-year-old woman presented to our emergency department due to a 1-week history of progressive confusion, personality change, vertigo, and stroke-like episodes with temporary aphasia. Clinical examination revealed fine motor impairment of the left hand, bilateral pyramidal signs, and deficits in memory, attention, and executive functions. As the patient was agitated, only a brief magnetic resonance imaging (MRI) protocol (diffusion-weighted, T2 and fluid attenuated inversion recovery [FLAIR] sequences) was performed, revealing multiple supra- and infratentorial punctate diffusion-restricted lesions as well as FLAIR-hyperintense white matter lesions without evidence of restricted diffusion. In addition, the central corpus callosum revealed round (*snowball*), linear (*spoke*), and “hanging” (*icicles*) T2 and FLAIR-hyperintense lesions (Fig. 1, Panel A). Due to these pathognomonic imaging findings, Susac Syndrome, an immune-mediated endotheliopathy of precapillary arterioles of the brain, retina, and inner ear [1], was considered as the most likely differential diagnosis. The patient was admitted to our neurological intensive care unit for observation and further diagnostic workup until potentially life-threatening differential diagnoses had been excluded. Sedation or mechanical ventilation was not necessary.

## Discussion of Differential Diagnoses

Cerebrospinal fluid (CSF) analysis revealed a normal cell count, which made an infectious etiology unlikely. Still, she was treated with intravenous aciclovir (10 mg/kg bodyweight every 8 h) for 24 h until negative results for herpes virus polymerase chain reaction were obtained. CSF protein was mildly elevated (98 mg/dl, normal range < 60 mg/dl), indicating dysfunction of the blood–brain barrier, and intrathecal immunoglobulin synthesis was detected. Screening for antibodies against neuronal surface and intracellular antigens as well as for thyroid antibodies was negative, arguing against the differential diagnosis of autoimmune encephalitis or encephalopathy including steroid-responsive encephalopathy associated with autoimmune thyroiditis. Based on the clinical presentation and neuro-imaging findings (lesions in the central corpus callosum), as well as the pronounced dysfunction of the blood–brain barrier (indicated by the highly elevated CSF-to-serum albumin ratio > 47), the differential diagnosis of MS was unlikely.

After proper treatment of the agitated state, an extended MRI protocol with susceptibility-weighted sequences and contrast-enhanced angiography was performed but did not reveal vascular abnormalities or signs of hemosiderin deposition. A thorough screening for other organ manifestations including systemic vasculitis was uneventful. Despite the lack of biopsy results, CNS vasculitis was therefore not considered as plausible differential diagnosis. Uneventful transesophageal echocardiography and laboratory screening for antiphospholipid syndrome and other coagulopathies made juvenile stroke unlikely.

The diagnosis of Susac syndrome was finally confirmed by retinal fluorescein angiography (Fig. 1, Panel B), showing multiple branch retinal artery occlusions and arterial wall hyperfluorescence, and by pure-tone audiometry revealing hearing loss for low- and mid-tone frequencies. After high-dose intravenous methylprednisolone therapy (1 g/day for 3 days) and intravenous immunoglobulin administration (2 g/kg bodyweight over 2 days), neuropsychological and motor symptoms markedly improved. Treatment was continued with oral methylprednisolone (1 mg/kg bodyweight with slow tapering), mycophenolate mofetil (2 g/day), and intravenous immunoglobulins (1 g/kg bodyweight every other week). After 3 months (five cycles of intravenous immunoglobulins), the patient had fully recovered except for mild hearing deficits. Neuro-imaging did not reveal new lesions. Therefore, so far, no treatment intensification in terms of adding tacrolimus, cyclophosphamide, or rituximab was necessary.

It is important to mention that the patient never suffered from headache and CSF examination revealed intrathecal immunoglobulin synthesis, which is both not typical for Susac syndrome and made a broad laboratory and neuro-imaging workup necessary.

## Conclusion

Susac Syndrome is a rare but probably under-recognized condition typically manifesting with headache, encephalopathy, and focal neurological signs. It occurs primarily in young women (3–4 times more often than in men) at a median age of 30–35 years at onset [2] and affects the microvasculature of the brain, retina, and inner ear. Pathophysiologic mechanisms are incompletely understood; however, histopathologic findings and clinical improvement after initiation of immunosuppressive therapy strongly suggest an immune-mediated mechanism. The full diagnostic triad consists of (1) neurological manifestations and typical MRI findings (brain involvement), (2) branch retinal artery occlusions or arterial wall hyperfluorescence in retinal fluorescein angiography or signs of retinal branch ischemia in fundoscopy or optical coherence tomography (retinal involvement), and (3) new tinnitus, hearing loss or peripheral vertigo (cochlear involvement). Recommended treatment includes corticosteroids, intravenous immunoglobulins, and mycophenolate mofetil [3]. Depending on disease severity, tacrolimus, cyclophosphamide, or rituximab may be added. Treatment for at least 2 years is recommended [3]. Important differential diagnoses include MS, CNS vasculitis, autoimmune encephalopathies, and juvenile stroke.

## References

[1] Kleffner I, Dorr J, Ringelstein M (2016). Diagnostic criteria for Susac syndrome. J Neurol Neurosurg Psychiatry.

[2] Seifert-Held T, Langner-Wegscheider BJ, Komposch M (2017). Susac’s syndrome: clinical course and epidemiology in a Central European population. Int J Neurosci.

[3] Rennebohm RM, Asdaghi N, Srivastava S, Gertner E (2018). Susac’s syndrome: clinical course and epidemiology in a Central European population. Int J Stroke.

